# Expert Views on HPV Infection

**DOI:** 10.3390/v10020094

**Published:** 2018-02-24

**Authors:** Alison A. McBride, Karl Münger

**Affiliations:** 1 Laboratory of Viral Diseases, National Institute of Allergy and Infectious Diseases, 33 North Drive, MSC3209, National Institutes of Health, Bethesda, MD 20892, USA; 2 Department of Developmental, Molecular and Chemical Biology, Tufts University School of Medicine, Boston, MA 02111, USA

The goal of this Special Issue was to obtain expert viewpoints on unresolved, controversial or emerging topics related to the natural history, evolution, biology, and disease association of HPV infection. The resulting articles cover a wide range of thought provoking topics.

There are over four hundred different papillomavirus (PV) types, which replicate in mucosal and cutaneous stratified epithelial surfaces giving rise to a wide range of lesions. Papillomaviruses have a remarkable life style that relies on the differentiation state of the host epithelium; they infect the basal cells of the epithelium and establish a quiescent infection in the proliferative cells. As the infected cells differentiate, the productive life cycle is activated, and viral-laden squames are eventually released from the surface of the epithelium. To support this life style, PVs interact with, and manipulate, many key cellular pathways. In this Issue, Puustusmaa and colleagues present an intriguing study in which they searched the biosphere for distant homologs of PV protein domains in a quest to discover the origin of papillomaviruses [1]. Suarez and Travé describe insights obtained from a review of PV E6 and E7 structural data [2] and Campos reviews the remarkable abilities of the minor capsid protein L2 to deliver the viral genome to the nucleus upon infection [3]. Moody describes how PVs interface with signaling pathways to provide the virus with a replication-competent environment in differentiating cells [4], and Graham describes how PV late gene expression is regulated by keratinocyte differentiation [5]. MmuPV1, a virus capable of infecting laboratory strains of mice was first described in 2011, and Hu et al. review the remarkable progress made using this valuable model [6]. 

Over three hundred human papillomavirus (HPV) types have been described and HPV infection is ubiquitous. However, many questions remain about infection, progression and resolution of HPV-associated disease. Gravitt and Weiner present a natural history model across the lifespan of an infected individual, with a particularly focus on the role of viral latency [7]. Alizon and colleagues review our current knowledge about acute/transient infections to provide insight as to why some infections are efficiently cleared while others become persistent [8]. The article by Herfs et al. explains why mucosal junction cells in epithelial transition zones are particularly susceptible to HPV infection and carcinogenic progression [9], while Spurgeon and Lambert describe the role of the stroma and microenvironment in these processes [10]. Continuing in this theme, Strati reviews the role of stem cell dynamics in HPV infection [11]. 

A subset of alpha-HPVs are oncogenic and are the causative agent of approximately 5% human cancers. Viral manipulation of host pathways can inadvertently promote oncogenesis and several articles in the Special Issue address this. Katzenellenbogen describes the role of telomerase activation in HPV infection and oncogenesis [12], while Warren and colleagues discuss the role of APOBEC3 induction in these processes [13]. Guenat et al. review recent studies showing that HPV regulates the content of exosomes and discuss how this might promote carcinogenesis [14]. Khoury and colleagues explain why the study of HPV infection in individuals prone to cancer due to mutations in DNA repair pathways provides an opportunity to uncover viral and host susceptibility factors [15]. Mirabello et al. report on a meeting of HPV experts that convened to discuss the intersection of HPV epidemiology, genomics and mechanistic studies of HPV-mediated cervical carcinogenesis [16]. Only HPVs from the alpha genus have been officially declared carcinogenic, but there is much discussion about the potential role of beta-HPVs in the initiation of non-melanoma skin cancer. Hufbauer and Akgül describe beta-HPV oncogenic mechanisms that may be relevant for the development of skin cancer [17].

HPV-associated cancers acquire profound changes and phenotypes that are important for carcinogenesis and could impact prognosis and treatment. Morgan and colleagues reevaluate the status of integrated and extrachromosomal HPV genomes in head and neck cancer [18] and Litwin et al. review somatic cell mutations that frequently occur in HPV-driven cancers [19]. Soto and colleagues review epigenetic alterations in HPV-associated cancers and explain why these reversible modifications might be amenable to epigenetic therapy [20]. Hoppe-Seyler et al. describe how many HPV-associated cancers have regions of hypoxia containing dormant cancer cells with no viral oncogene expression and explain why this has important consequences for treatment [21]. Finally, two articles review how HPVs modulate factors and pathways important for viral persistence and discuss therapies that could target these key processes. Shanmugasundaram and You describe the mechanisms required for viral genome persistence and discuss how small molecule therapeutics could disrupt this process [22]. Smola reviews the complex interplay between HPV-infected cells and the local immune microenvironment and discusses the potential of related diagnostics and immunotherapies [23]. 

We thank the authors and reviewers for giving their time, and sharing their expertise, to contribute to this stimulating collection of articles. We hope that the Special Issue has provided insight into many aspects of HPV infection and will inspire future questions, ideas and research. 
